# Exertional Rhabdomyolysis, Hyposthenuria, and Acute Kidney Injury: The Non-benign Side of Sickle Cell Trait

**DOI:** 10.7759/cureus.103115

**Published:** 2026-02-06

**Authors:** Ashley C Vincent, Irmina Swiostek, Rehaan Shaffie

**Affiliations:** 1 Internal Medicine, University of Colorado Anschutz, Aurora, USA; 2 Internal Medicine, Rush University Medical Center, Chicago, USA; 3 Hospital Medicine, Denver Health, Denver, USA

**Keywords:** dehydration, hyperkalemia, hyposthenuria, renal failure, rhabdomyolysis, sickle cell trait

## Abstract

Sickle cell trait (SCT) is largely understood to be a clinically silent disease that typically does not require intensive clinical monitoring or counseling of patients. In fact, many patients with SCT are unaware that they have this genetic condition. However, emerging studies, case reports, and reviews increasingly demonstrate that severe clinical pathology can be associated with SCT, showcasing the need for improved counseling and education. We present the case of a healthy young male patient who was admitted to the hospital with rhabdomyolysis, acute liver injury, extreme electrolyte disturbances, and acute renal failure necessitating emergent hemodialysis. Given that this was an otherwise healthy young athlete with no known risk factors, the gravity of his clinical condition led our team to question why he had such a severe presentation. Further evaluation revealed the diagnosis of SCT. SCT has been linked to an increased risk of exertional rhabdomyolysis, which causes muscle damage via microvascular occlusion as well as tissue ischemia, caused by endothelial damage. These processes predispose to a decreased ability to concentrate urine, increasing risk for dehydration, and more serious clinical presentations. The potential links between SCT and exertional rhabdomyolysis support the hypothesis that SCT is not a clinically silent condition.

## Introduction

Sickle cell trait (SCT) has historically been interpreted as a genetic finding with minimal clinical significance. Unlike its homozygous counterpart, SCT is a heterozygous condition that has been associated with a few disease states, in which patients may never know they have it or remain asymptomatic throughout their lives. However, SCT is a prevalent condition in the world, and its prevalence is highest amongst individuals of African-American descent [1,2]. There are well-known associations between SCT and clinically significant disease states, particularly related to the kidney, i.e., chronic kidney disease, proteinuria, hematuria, and more rarely, renal medullary carcinoma [3]. SCT is now increasingly recognized as a predisposing risk factor for exertional rhabdomyolysis [2,3], with emerging data suggesting an association between SCT and severe acute kidney injury (AKI) [4]. Furthermore, there is now a more established relationship between SCT and an inability to concentrate urine (hyposthenuria), which may further increase the risk of dehydration, exertional rhabdomyolysis, and acute renal failure [3,4].

This is a case report of a patient who was found to have SCT incidentally after an athletic competition when he presented with acute renal failure. We explore the correlations between SCT, exertional rhabdomyolysis, hyposthenuria, and acute renal injury. 

## Case presentation

A previously healthy 24-year-old African-American male patient presented to the emergency room with severe myalgias, weakness, and bilateral lower extremity numbness, two days after a week of intensive exercise with minimal hydration. He initially presented to another hospital, received an MRI of his lumbar spine, which was unrevealing, and was discharged home with muscle relaxants. He re-presented two days later to our hospital with increased bilateral lower extremity pain, numbness, weakness, and dark urine. Vital signs and physical exam were unremarkable, other than mild hypertension, non-focal diffuse weakness, and a mild hyperpigmented rash on the dorsum of his hands and feet. His presenting labs included a complete blood count, complete metabolic panel and creatine kinase, and urinalysis (Tables 1-3).

**Table 1:** Complete blood count depicting absence of anemia INR: International Normalized Ratio; PT: Prothrombin Time.

	Patient’s result	Reference range
White blood count (k/mL)	30.6	4.5-10.0
Hemoglobin (g/dL)	19.5	13.0-18.0
Hematocrit (%)	53.5	38.0-52.0
Platelets (k/mL)	293	150-400
INR	1.21	0.83-1.19
PT (seconds)	15.4	11.4-15.2

**Table 2:** Comprehensive metabolic panel depicting evidence of renal injury, electrolyte disturbances, and liver injury BUN: Blood Urea Nitrogen; eGFR: Estimated Glomerular Filtration Rate; AST: Aspartate Aminotransferase; ALT: Alanine Aminotransferase.

	Patient’s result	Reference range
Sodium (mmol/L)	122	135-143
Potassium (mmol/L)	8.6	3.6-5.1
BUN (mg/dL)	63	6-22
Creatinine (mg/dL)	6.03	0.50-1.39
eGFR (mL/min/1.73m^2^)	12.48	>60.00
Calcium (mg/dL)	5.7	8.1-10.5
Phosphorus (mg/dL)	13.9	2.7-4.8
AST (U/L)	8,420	<40
ALT (U/L)	2,506	7-45
Alkaline Phosphatase (U/L)	91	35-137
Creatine Kinase (U/L)	51,100	39-308

**Table 3:** Urinalysis results depicting evidence of myoglobinuria RBC: Red Blood Cell; WBC: White Blood Cell.

	Patient’s results	Reference range
Color	Brown	Colorless, light yellow, yellow, dark yellow
Clarity	Cloudy	Clear
Specific gravity	1.025	1.001-1.035
pH	6.0	5.0-8.0
Leukocytes	1+	Negative
Nitrite	Positive	Negative
Protein	3+	Negative
Glucose	2+	Negative
Ketones	Trace	Negative
Blood	3+	Negative
RBC	>100	None seen, 0-1, 2-5 /HPF
WBC	11-20	None seen, 0-1, 2-5 /HPF
Squamous epithelial	Few	None seen /HPF
Myoglobin (mg/L)	19	0-1

He presented with a concentrated appearing complete blood count (Table 1), severe hyperkalemia, acute renal injury, and evidence of acute liver injury (Table 2). Urinalysis yielded a dark sample with elevated red blood cells and urine myoglobin.

After several unsuccessful strategies at improving the hyperkalemia, and peaked T waves on ECG suggesting cardiac instability, the patient underwent emergent dialysis. Throughout his week-long hospital stay, he remained on intermittent hemodialysis with little to no sign of renal recovery. Given the severity and unclear nature of his severe presentation, including acute liver injury and a hyperpigmented rash, the medical ICU providers consulted gastroenterology and rheumatology specialists to ensure an underlying autoimmune etiology of his presentation was not missed. Through further diagnostics and monitoring, no evidence of other etiology of acute liver injury or autoimmune pathology (i.e. myopathy) was discovered.

A screen for sickle cell disease was performed by the emergency room providers, possibly given his acute pain leg pain and numbness, as well as mentioned family history of SCT. Hematology was consulted by the primary ICU team when the routine sickle cell screen gave positive results, and recommended a confirmatory hemoglobin electrophoresis and a peripheral smear which ruled out sickle cell disease and later revealed the diagnosis of SCT. His hyperpigmented rash was attributed to a systemic inflammatory reaction which improved with intravenous fluids and dialysis. He was discharged home on outpatient hemodialysis, and his renal function began to improve a week after discharge. 

## Discussion

SCT is generally understood to be a clinically benign condition, especially when compared to its homozygous counterpart, sickle cell disease. SCT is a heterozygous condition in which individuals inherit one sickle cell gene and one normal gene. Under typical circumstances, and even in instances of low-to-moderate physiological stress, SCT generally does not result in clinically significant sickling [1-3]. However, in circumstances of more severe cellular stress (i.e., acidosis, dehydration), clinically detrimental health outcomes, such as exertional rhabdomyolysis and acute renal failure, can occur [3-6]. In our case presentation, exertional rhabdomyolysis led to a severe, sustained AKI with subsequent renal failure, necessitating hemodialysis. 

There are numerous case reports and multiple studies, dating back to the 1970s, documenting a link between SCT and an increase in patients’ risk of exertional rhabdomyolysis [1-2,7-11]. A large 2016 retrospective cohort study of US Army soldiers determined that there was a significantly higher risk of exertional rhabdomyolysis among soldiers with SCT [2]. A 2018 systematic review found a positive direct correlation between SCT and exertional rhabdomyolysis [1]. Given this, the association between SCT and exertional rhabdomyolysis is now more commonly known. The exact mechanism of why those with SCT are at increased risk of exertional rhabdomyolysis is not completely understood, though some reports have theorized that the pathophysiology may include exertional sickling [8,9,12]. The sickling process in SCT is not nearly as common as in sickle cell disease, and typically only occurs in periods of extreme physiologic stress [13]. Rhabdomyolysis results from increased cytoplasmic and mitochondrial calcium. This then releases proteases and causes cellular dysfunction, and results in the depletion of intracellular adenosine triphosphate (ATP) and/or direct rupture of cell membranes [14,15]. This cellular breakdown leads to severe pain, myoglobinuria, rising creatine kinase levels, electrolyte disturbances, and in some cases, acute renal failure [14,16]. The renal failure in patients with rhabdomyolysis is thought to be secondary to the nephrotoxic myoglobin and acid production released from the breakdown of muscle tissue, as well as hypoperfusion to the kidneys [17]. This coupled with possible exertional sickling with SCT, increases the risk of severe AKI significantly.

In the adult population, common causes for rhabdomyolysis include acute illness or infection, alcohol, illicit drugs, medications, surgery, trauma or crush injury, and exercise [15]. Various studies have linked underlying risk factors for rhabdomyolysis (either traumatic or non-traumatic) to male sex, dehydration, body mass index over 40, longstanding use of lipid-lowering medications, age extremes (age less than 10 years or over 60 years), to name only a few [15]. Exertional rhabdomyolysis does not have a formal definition though it is typically diagnosed by elevated creatine kinase and muscle tenderness/pain after recent vigorous exercise (without recent trauma or other direct injury) [16]. Non-traumatic exertional rhabdomyolysis is even more likely to occur when the exercise is prolonged, eccentric exercises, hot and humid environments, and/or increased sweating [14]. SCT was not previously known to be a risk factor for exertional rhabdomyolysis, though it is now more commonly known as a risk factor for muscle breakdown.

This male patient was previously healthy, young, and athletic; his only known non-modifiable risk factors for rhabdomyolysis (at the time of presentation) were male gender and Black race. His only modifiable risk factors were dehydration and an intense athletic competition the week prior. Due to the severity of his presentation, which included not only renal failure, but also acute liver injury, a broad diagnostic evaluation of autoimmune conditions, toxic ingestions, and myopathy-related causes were investigated, though no alternate etiologies were discovered. Given his severe muscle pain and mention of family history of sickle cell disease or SCT, a screen for sickle cell disease was also performed on presentation and was positive. Hematology was consulted, though he had lower suspicions for sickle cell disease given his lack of anemia, and no previous presentations for acute pain episodes or complications of sickle cell disease. Hemoglobin electrophoresis was therefore performed to rule out sickle cell disease, and in turn, revealed a diagnosis of SCT. Due to the severity of rhabdomyolysis and the multi-organ involvement, our team questioned whether this diagnosis of SCT played a role in his clinical outcomes, specifically whether it could lead to an increased risk of not only exertional rhabdomyolysis but also acute renal failure. 

SCT at baseline has also been hypothesized to be associated with an increased risk of chronic kidney disease, a faster decline in glomerular filtration rate (GFR), and an increased risk of AKI, specifically severe and sustained AKIs [6,18]. It is also well established that those with SCT are at increased risk of hyposthenuria and have an impaired ability concentrate their urine [3]. The concentration of urine occurs in the renal medulla, which is a sensitive and delicate region of the kidney, made up of the vasa recta, tubules, and thin inter-tubule capillaries [3,19]. Under times of severe stress, (i.e., acidosis, nephrotoxic proteins) damage to the medulla can occur, which leads to a reduction in its concentrating capacity leading to further dehydration and renal dysfunction [3]. Previous case reports and articles have alluded to a potential link between hyposthenuria and the severity of the rhabdomyolysis [9]. Due to the organ failure on presentation we questioned whether SCT led to an increased risk of acute renal injury. 

In those with SCT, there is an underlying risk of hyposthenuria, which leads to dehydration through excess water losses in the urine, leading to further dehydration, and facilitates the development of acute renal injury [18]. This is, in part, due to the low flow state of the medulla, which has been postulated as an ideal condition for sickled hemoglobin S (HbS) to form further exacerbating hyposthenuria [3,5]. The combination of dehydration, inability to concentrate urine, and physiologic stress induced by severe rhabdomyolysis, all likely led to the severe renal presentation seen in this patient. Given this, adequate hydration and patient education regarding risks with intense physical activity may be key factors in preventing severe renal failure and dehydration that occurs in those with SCT. 

In clinical practice, identification of those at risk for both exertional and non-exertional rhabdomyolysis is important so that patients may be appropriately counseled. It may be difficult to identify patients who have risk factors for rhabdomyolysis beyond medications they are using, though baseline counseling for dehydration, especially those in athletic occupations, competitions, or those new to exercise may be beneficial. Younger patients who have a family history of sickle cell disease may also benefit from SCT screening, as this may guide further education regarding future risks, a thorough family history may prove beneficial in this setting. It has been hypothesized that patients of African-American descent may be at an even higher risk, and studies have shown that this may be related to enzymatic deficiencies and a higher creatine kinase to coenzyme Q ratios [20]. Therefore, African-American patients may benefit from screening for SCT and clinical education surrounding exertional rhabdomyolysis risks to prevent life-threatening organ failure.

## Conclusions

This report adds to the existing literature about the clinically significant outcomes associated with SCT and posits several possible mechanisms for why these occur. Limitations of this case report include its single-case nature and the fact that the patient was not screened or tested for other enzymatic or genetic deficiencies that may have led to his complex and grave presentation. However, we do recommend counseling patients with SCT about the risks of exertional rhabdomyolysis, specifically the risks of dehydration during times of moderate-to-intense exercise. Further, those with SCT may be particularly vulnerable to more severe acute renal failure in these times of cellular stress due to underlying hyposthenuria and the decreased ability to concentrate urine. In the primary care setting, it may be beneficial to obtain a thorough family and exercise history, especially in younger male patients.
